# Diagnosis‐based emergency department alcohol harm surveillance: What can it tell us about acute alcohol harms at the population level?

**DOI:** 10.1111/dar.12458

**Published:** 2016-10-27

**Authors:** Genevieve Whitlam, Michael Dinh, Craig Rodgers, David J. Muscatello, Rhydwyn McGuire, Therese Ryan, Sarah Thackway

**Affiliations:** ^1^ Centre for Epidemiology and EvidenceNSW Ministry of HealthSydneyAustralia; ^2^ Emergency DepartmentRoyal Prince Alfred HospitalSydneyAustralia; ^3^ Alcohol and Drug ServiceSt Vincent's HospitalSydneyAustralia; ^4^ School of Public Health and Community MedicineUniversity of NSWSydneyAustralia

**Keywords:** alcohol‐related disorder, intoxication, emergency department, public health surveillance, triage

## Abstract

**Introduction and Aims:**

Acute harm from heavy drinking episodes is an increasing focus of public health policy, but capturing timely data on acute harms in the population is challenging. This study aimed to evaluate the precision of readily available administrative emergency department (ED) data in public health surveillance of acute alcohol harms.

**Design and Methods:**

We selected a random sample of 1000 ED presentations assigned an ED diagnosis code for alcohol harms (the ‘alcohol syndrome’) in the New South Wales, Australia, automatic syndromic surveillance system. The sample was selected from 68 public hospitals during 2014. Nursing triage free‐text fields were independently reviewed to confirm alcohol consumption and classify each presentation into either an ‘acute’ or ‘chronic’ harm. Positive predictive value (PPV) for acute harm was calculated, and predictors of acute harm presentations were estimated using logistic regression.

**Results:**

The PPV of the alcohol syndrome for acute alcohol harm was 53.5%. Independent predictors of acute harm were ambulance arrival [adjusted odds ratio (aOR) = 3.4, 95% confidence interval (CI) 2.4–4.7], younger age (12–24 vs. 25–39 years: aOR = 3.4, 95% CI 2.2–5.3), not being admitted (aOR 2.2, 95% CI 1.5–3.2) and arriving between 10 pm and 5.59 am (aOR 2.1, 95% CI 1.5–2.8). PPV among 12 to 24‐year‐olds was 82%.

**Discussion and Conclusions:**

*The alcohol syndrome provides moderate precision as an indicator of acute alcohol harms presenting to the ED. Precision for monitoring acute harm in the population is improved by filtering the syndrome by the strongest independent predictors of acute alcohol harm presentations.* [Whitlam G, Dinh M, Rodgers C, Muscatello DJ, McGuire R, Ryan T, Thackway S. Diagnosis‐based emergency department alcohol harm surveillance: What can it tell us about acute alcohol harms at the population level? *Drug Alcohol Rev* 2016;35:693–701]

## Introduction

It is estimated that alcohol misuse cost the Australian society $14.4 billion in 2010, of which almost $1.7 billion was to the health system 1. In 2013, 26% of people aged 14 years or older consumed five or more standard drinks on a single drinking occasion at least once a month, placing them at risk of an alcohol‐related injury 2, 3. With an estimated 4–14% of emergency department (ED) presentations in Australia involving excessive alcohol consumption, depending on the time of the week, the ED remains a key setting in responding to alcohol harms 4, 5, 6. Such harms range from acute intoxication and poisoning to life‐threatening events due to intoxication (e.g. respiratory distress), injuries from alcohol‐related accidents and assaults and chronic alcohol misuse problems (e.g. withdrawal) 3.

Capturing timely, accurate and useful ongoing data on people presenting to the ED with alcohol‐related harms are required to monitor changes over time, guide planning and evaluation of policy and support health service delivery. However, in Australia, this remains a challenge, as it is not mandatory to screen for or collect data on alcohol‐related ED presentations 4. Typically, the policy response relies on research from cross‐sectional or longitudinal survey‐based studies to estimate the prevalence of alcohol‐related ED presentations 4, 5, 7, 8. However, these study designs can be costly, are limited in their generalisability and are not timely enough to result in changes to service delivery. Their design often does not allow estimation of the impact of extreme drinking leading to acute hospital care. Considerable research has also been conducted on alcohol‐related injury ED presentations, using surveys or ED injury surveillance data 9, 10, 11, 12. However, these studies do not capture people who present with alcohol‐related harms unrelated to injuries, such as alcohol poisoning.

Continuous syndromic surveillance in EDs in New South Wales (NSW), Australia, was established in 2003 and is used to rapidly identify increases in public health harms, including those due to infectious diseases, injury and alcohol and other drugs 13, 14, 15. This surveillance system provides a near real‐time, ongoing data feed of ED presentations. Syndromes are created by automatically grouping related clinician‐coded ED diagnosis information. Electronic access to two free‐text nursing fields is available for various purposes, including validation of syndrome groups. Evidence suggests that syndromes provide reasonable accuracy or correlation with community disease incidence 13, 15.

The alcohol syndrome is one of nine broad syndrome categories. It was developed for public health surveillance of alcohol harms at the population level and to describe alcohol harms occurring during major events or mass gatherings, such as New Year's Eve 6, 16, 17, 18, 19, 20, 21. To achieve this purpose, the syndrome is composed of a series of alcohol harm ED diagnosis codes, including codes for intoxication, mental and behavioural disorders, gastritis, poisoning, dependence, withdrawal, rehabilitation and counselling and evidence of alcohol in the blood. Blood alcohol testing codes are excluded, as they do not necessarily represent alcohol harms. The codes available in the various ED information systems used in NSW do not include complete diagnostic classifications and are not formally implemented with formal coding in mind. Thus, the codes were selected using a combination of both relevant codes gleaned from classifications and analysis of codes actually available and used in the various information systems.

Applications of administrative ED data are limited, as it is not collected or designed to answer specific research or policy‐relevant questions. The diagnosis codes available in ED information systems are influenced by the software system used and do not necessarily reflect the intent of those codes from formal health classifications. Also, ED information systems are not designed to record alcohol as a contributing factor in the ED presentation. For instance, diagnostic codes in the alcohol syndrome only capture around 24% of all alcohol‐related ED presentations because many alcohol‐related ED presentations are coded as other problems, such as injury, or they leave before treatment and diagnosis could occur 18.

In the absence of otherwise readily—and routinely—available data, the alcohol syndrome in the NSW ED surveillance system provides an opportunity to monitor trends in ED presentations for alcohol harms at the population level, across both time and place in near‐real time 16, 18, 19, 20, 21, 22. However, detailed understanding of the extent to which it captures acute and chronic alcohol harms is needed to clarify its policy and epidemiological relevance.

The United States Centers for Disease Control and Prevention identifies positive predictive value as an important attribute of a surveillance system 23. The objective of the present study was to evaluate the positive predictive value of coded alcohol ED presentations to identify acute alcohol harm presentations. Additionally, it aimed to determine factors associated with acute alcohol harm presentations, which may guide or improve application of the syndrome and interpretation of trends and epidemiological information obtained using the syndrome.

## Methods

### Data source

This study used data from the NSW ED surveillance system 24. The 68 hospitals included in this study provided a continuous data feed to this system in 2014 and represented approximately 85% of ED activity in NSW. The data feeds are drawn from established patient management and electronic medical record information systems used at the hospitals.

This surveillance system uses syndromes, which are automatically grouped ED presentations based on the diagnosis classification allocated from the mandatory provisional ED diagnosis field assigned by the treating clinician at patient discharge or admission to a hospital ward. Diagnosis terms selected by the clinician are mapped to a code by the hospital information system. The codes used include any of the Australian clinical implementations of the International Classification of Diseases (ICD) 9th revision, ICD‐10th revision (ICD‐10‐AM) or the Systematized Nomenclature of Medicine—Clinical Terminology (SNOMED CT) 25, 26, depending on the system used at the hospital. The syndromes are built on ICD‐10‐AM codes. Mappings made available by the National e‐Health Transition Authority with the introduction of SNOMED‐CT in Australia were used to select SNOMED‐CT concept identifiers corresponding to relevant ICD‐10‐AM codes. In establishing syndrome definitions for the surveillance system, diagnosis descriptions actually occurring in the ED information system records were also analysed to determine other relevant SNOMED‐CT concept identifiers. ICD‐9‐CM codes were mapped to ICD‐10‐AM codes, according to the National Centre for Classification in Health 27.

### Population

The population for this study was defined as all presentations to 68 NSW EDs recorded in the surveillance system in 2014 that were grouped into the alcohol syndrome (see Table S1 for included codes). This syndrome includes diagnostic codes for acute alcohol harms (e.g. heavy episodic drinking), chronic alcohol problems (e.g. alcohol withdrawal syndrome) and general alcohol use (e.g. current drinker of alcohol). It does not identify presentations where alcohol may have contributed to a presentation, but was not coded as the ED diagnosis, such as alcohol‐related injuries.

Sample size calculations were based on the precision to detect the proportion of patients who were coded as an acute alcohol harm presentation. Based on advice from surveillance officers who routinely use the surveillance system, it was estimated that 60% of the records in the alcohol syndrome represented acute alcohol harms. Using this estimate, a sample size of 1000 was calculated to be sufficient to provide a relative standard error of 2.5 at the 95% confidence level. The sample was selected using a simple random sample method in Statistical Analysis System (SAS) version 9.4 for Windows.

Information provided for each ED presentation included patient demographics (age, sex, socio‐economic status and remoteness area of patient residence), diagnostic information (diagnosis code and description), service delivery characteristics (hospital facility, location of hospital, arrival date and time, mode of arrival, triage category and departure status) and two de‐identified free‐text fields recorded by triage nurses which describe the presenting problem and nursing assessment.

### Manual review of the triage nursing text

Manual review of the triage nursing text was conducted for the following purposes:

1. Confirmation of alcohol consumption (GW)

An ‘alcohol’ flag was applied to each record that referred to:

- generic terms, including ‘alcohol’ and known abbreviations (e.g. ‘ETOH’ or ‘booze’);
- type of alcohol, including colloquial language (e.g. beer and wine);
- consumption terms (e.g. ‘drinking’);
- intoxication terms (e.g. ‘inebriated’ or ‘drunk’);
- amount terms (e.g. ‘pint’ or ‘cask’); and
- location of consumption (e.g. pub or club) 18, 28.

The flag was not applied to a record if the patient denied alcohol consumption.

Classification of each record into ‘type of alcohol harm’ (dependent variable) (MD, CR and GW)

Classification was conducted by three independent reviewers: an ED physician, an addiction medicine specialist and a psychologist. Two free‐text nursing triage assessment fields were reviewed to classify each record into one of four alcohol harm types: acute alcohol intoxication, chronic alcohol misuse, acute alcohol intoxication in a person with a chronic alcohol misuse problem and undetermined (definitions provided in Table S2). Reviewers were blinded to all other fields during this process. In the event of disagreement, the final decision was made by majority rule or discussion with the aim of reaching a consensus or majority rule.

Identification of co‐morbid factors (independent variables) (GW)

A flag was assigned against each record that referred to co‐morbid factors, including mental health problems, current suicide or self‐harm attempt or ideation, current poly‐substance use, injury or police involvement. If noted, details of poly‐substance use, type of injury (violent or non‐violent) and the Glasgow Coma Scale (GCS) score were recorded.

### Statistical analysis

All statistical analyses were performed using SAS. The dependent variable for the logistic regression analysis was coded as a dichotomous variable, with 1 being equal to ‘acute alcohol intoxication’ presentations (known as ‘acute’ throughout) and 0 being equal to the combined group of chronic alcohol misuse, acute alcohol intoxication in a person with a chronic alcohol misuse problem and undetermined (known as ‘chronic’ throughout). This grouping was intended to ensure that the dependent variable of interest captured acute alcohol intoxication harms only (as a proxy for binge drinking), as distinct from the population that may have an underlying chronic alcohol problem. This is because: (i) the population with an underlying chronic alcohol problem require different, more intensive interventions than the population who present with harms related to binge drinking 29; (ii) recent policy and legislative changes are focused on reducing binge drinking and related harms; and (iii) it allowed for a more conservative analysis.

Descriptive statistics were used to compare acute with chronic alcohol harm ED presentations. Univariate logistic regression was conducted to determine statistically significant differences between the groups and to quantify these differences. Backward stepwise selection was used to determine the variables to be included in the final multivariate logistic regression model. Factors with a univariate logistic regression Wald test *P*‐value cut‐off point of 0.25 were included in the initial multivariate logistic regression model. Covariates remained in the model as a confounder if their exclusion resulted in a change in any other parameter estimate by 15% or more 30. The positive predictive value was defined as the proportion of alcohol‐coded ED presentations that were classified as acute harm. To check for clustering at the hospital level, a sensitivity analysis was performed using multivariate logistic regression with generalised estimating equations. This did not identify evidence of clustering.

### Ethics

Ethical approval was granted by the NSW Population and Health Services Research Ethics Committee (HREC/15/CIPHS/13).

## Results

### Sample characteristics

Of all the coded alcohol ED presentations, over one‐quarter were aged 12–24 years (27%), including 7% aged 12–17 years. The majority were men (64%), resided in major cities (76%) and in the three most disadvantaged socio‐economic quintiles (64%; Table 1). Over half the presentations arrived on weekdays (61%), and half arrived between 6 am and 9.59 pm (55%). A majority of presentations arrived by ambulance (68%), were triaged as non‐urgent (87%) and were not admitted (78%). The mean GCS score was 13.6 (range: 3.0–15.0). However, it was only recorded in 37% of the coded alcohol presentations.

**Table 1:** Characteristics of acute compared with chronic alcohol harm ED presentations

		Acute alcohol harms		Chronic alcohol harms		Total	Unadjusted OR (95% CI)	*P*‐value
Alcohol syndrome ED presentations		*n*	%	*n*	%	%		
*Demographic characteristics*								
Sex	Female	215	59.4	147	40.6	36.2	1.5 (1.1–1.9)	<0.05
	Male	320	50.2	318	49.8	63.8	1	
Age group, years	12–24	225	82.1	49	17.9	27.4	4.8 (3.2–7.1)	<0.001
	25–39	121	49.0	126	51.0	24.7	1	
	40–54	118	38.4	189	61.6	30.7	0.7 (0.5–0.9)	<0.05
	55+	71	41.3	101	58.7	17.2	0.7 (0.5–1.1)	0.12
Remoteness of residence*	Major cities	365	53.1	323	47.0	76.3	0.9 (0.7–1.2)	0.51
	Regional and remote	119	55.6	95	44.4	23.7	1	
Socio‐economic status of residence*	Most disadvantaged (quintiles 3–5)	304	51.8	283	48.2	63.7	0.8 (0.6–1.1)	0.18
	Least disadvantaged (quintiles 1–2)	189	56.4	146	43.6	36.3	1	
*Service delivery characteristics*								
Location of ED	Metropolitan Sydney	383	52.0	353	48.0	73.6	0.8 (0.6–1.1)	0.12
	Rural and regional NSW	152	57.6	112	42.4	26.4	1	
Day of week	Weekend (Sat–Sun)	255	66.1	131	33.9	38.6	2.3 (1.8–3.0)	<0.001
	Weekday (Mon–Fri)	280	45.6	334	54.4	61.4	1	
Time of arrival	Late night (10 pm–5.59 am)	318	70.8	131	29.2	44.9	3.7 (2.9–4.9)	<0.001
	Daytime hours	217	39.4	334	60.6	55.1	1	
Arrival mode	Ambulance	415	61.1	264	38.9	68.0	2.7 (2.0–3.5)	<0.001
	Other	119	37.2	201	62.8	32.0	1	
Triage category	More urgent (1 and 2)	65	48.5	69	51.5	13.4	0.8 (0.5–1.1)	0.21
	Less urgent (3–5)	470	54.3	396	45.7	86.6	1	
Admission status	Not admitted	453	59.0	315	41.0	78.0	2.7 (1.9–3.7)	<0.001
	Admitted	76	35.0	141	65.0	22.0	1	
*Co‐morbidities*								
Current/past mental health problem	Yes	101	42.4	137	57.6	23.8	0.6 (0.4–0.7)	<0.001
	No	434	57.0	328	43.0	76.2	1	
Current suicide or self‐harm	Yes	50	46.7	57	53.3	10.7	0.7 (0.5–1.1)	0.14
	No	485	54.3	408	45.7	89.3	1	
Injury	Yes	126	70.8	52	29.2	17.8	2.4 (1.7–3.5)	<0.001
	No	409	49.8	413	50.2	82.2	1	
Type of injury	Violent	38	79.2	10	20.8	4.8	3.8 (1.9–7.8)	<0.001
	Non‐violent	73	68.9	33	31.1	10.6	2.2 (1.4–3.4)	<0.001
	Not specified	15	62.5	9	37.5	2.4	1.7 (0.7–3.9)	0.22
	No injury	409	49.8	413	50.2	82.2	1	
Current poly‐substance use	Yes	83	55.3	67	44.7	15.0	1.1 (0.8–1.5)	0.63
	No	452	53.2	398	46.8	85.0	1	
Police involvement or arrival	Yes	86	53.1	76	46.9	16.2	1.0 (0.7–1.4)	0.91
	No	449	53.6	389	46.4	83.8	1	
Total		535	53.5	465	46.5	100.0		

* Due to missing postcode or locality, overseas or interstate residence or no fixed/unknown address, 7.8% of records are missing socio‐economic status, and 9.8% are missing remoteness area. There is also <2% of presentations missing arrival mode or admission status.

CI, confidence interval; ED, emergency department; OR, odds ratio.

### Confirmation of alcohol consumption

Alcohol consumption was confirmed in 86% of the alcohol‐coded presentations. The remaining alcohol‐coded presentations (*n* = 138) either did not refer to alcohol consumption in the triage notes, or the triage fields indicated that the patient denied consuming alcohol.

### Distribution of alcohol harm types

The positive predictive value of the alcohol syndrome to identify acute harms was 54% (Table 1). Of the remaining presentations, 25% referred to persons with a chronic alcohol problem (14% chronic alcohol problem only; 12% acute alcohol intoxication in a person with a chronic alcohol problem). Around one‐fifth of the presentations could not be classified as acute or chronic (21%).

### Characteristics of acute presentations

**Figure 1:**
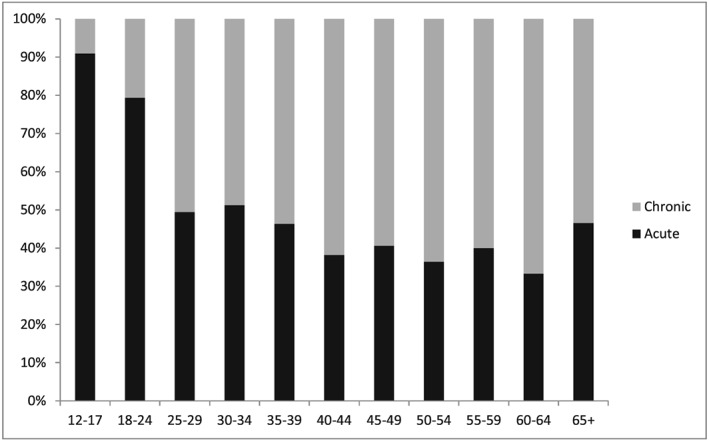
Distribution of acute and chronic alcohol harms to the emergency department, by age, NSW, 2014.

A large majority of coded alcohol presentations in people aged 12–24 years were for acute harms (82%; (Table 1). While only 7% of these presentations were by 12 to 17‐year‐olds, almost all from this age group presented with an acute harm (91%). In comparison, the proportion of acute harm presentations fluctuated between 33 and 51% for people aged 25 years and older (Figure 1). Acute harm presentations were relatively evenly spread between weekends (48%) and weekdays (52%). However, a majority presented to EDs between 10 pm and 5.59 am (59%), and many arrived by ambulance (78%). Yet, most were classified in non‐urgent triage categories (88%) and were not admitted (86%). Injuries were common (24%), with over half of these being non‐violent injuries, such as falls (14%).

The results from the unadjusted analysis show that sex, age, day of week, time of arrival, arrival mode, admission status, mental health problems and injury should be included in the initial multivariate model. Sex was not included in the final model, as it met the 0.15% significance level for exclusion.

### Independent predictors of acute alcohol harm ED presentations

Table 2 reports the adjusted differences between acute and chronic harm presentations. The findings indicate that after controlling for other factors, the strongest predictors of acute harm presentations were ambulance arrival [adjusted odds ratio (aOR) = 3.4, 95% confidence interval (CI) 2.4, 4.7], younger age (12–24 vs. 25–39 years: aOR = 3.4, 95% CI 2.2, 5.3) and late‐night arrival (aOR = 2.1, 95% CI 1.5, 2.8; Table 2). Presenting with an injury (aOR = 2.1, 95% CI 1.4, 3.2) and not being admitted (aOR = 2.2, 95% CI 1.5, 3.2) also remained independently associated with acute harm presentations.

**Table 2:** Adjusted odd ratios

Alcohol syndrome ED presentations		AOR (95% CI)	*P*‐value
Age group, years	12–24	3.4 (2.2–5.3)	<0.001
	25–39	1	
	40–54	0.7 (0.5–1.0)	0.07
	55+	0.8 (0.5–1.2)	0.21
Day of week	Weekday (Sat–Sun)	1.6 (1.2–2.2)	<0.05
	Weekend (Mon–Fri)	1	
Time of arrival	Late night (10 pm–5.59 am)	2.1 (1.5–2.8)	<0.001
	Daytime hours	1	
Arrival mode	Ambulance	3.4 (2.4–4.7)	<0.001
	Other	1	
Admission status	Not admitted	2.2 (1.5–3.2)	<0.001
	Admitted	1	
Mental health problem	Yes	0.7 (0.5–1.0)	0.08
	No	1	
Injury	Yes	2.1 (1.4–3.2)	<0.001
	No	1	

AOR, adjusted odds ratio; CI, confidence interval; ED, emergency department.

## Discussion

To our knowledge, this was the first study to quantify the precision of administrative data in capturing acute alcohol harms. The current indicator provides moderate precision at identifying acute alcohol harms presenting to the ED. Precision is improved by filtering the syndrome by the strongest independent predictors of acute alcohol harm presentations, such as younger age.

### Limitations

Just over one‐fifth of the coded alcohol ED presentations could not be classified as an acute or chronic harm, with many not referring to alcohol at all or the patient denying alcohol consumption. The presenting problems for these records included mental health problems, abdominal and chest pain, police requests for blood and urine tests and vomiting. These problems may or may not be related to alcohol consumption. For this group, it is possible that while the triage nurse did not assess alcohol to be involved or did not document alcohol involvement, the ED clinician assessed alcohol to be a primary presenting factor and coded the presentation as such. Miscoding is also possible, with previous studies reporting the validity of coded ED data to be susceptible to the unpredictable ED environment, varying staff coding competence, unintentional and intentional misclassification and patient–clinician communication problems 15, 31, 32. This group was included in the chronic harm group as they continue to be included in the routine operation of the system, and this ensures a more conservative analysis.

It is possible that our study underestimated chronic harms presenting to EDs due to insufficient detail in the triage notes or inadequate screening for chronic alcohol problems 33. To account for any potential underestimation, all undetermined presentations were combined with the chronic group.

Other known limitations of administrative ED data include variations between hospitals in: (i) text‐based discharge diagnosis options for clinicians; (ii) mapping of these text‐based discharge diagnosis options to coded diagnoses; and (iii) different coded classifications systems. In addition, the lack of standard questions on alcohol consumption in the ED and the variation in content and quality of triage notes limit reporting on alcohol consumption behaviour and subsequent outcomes.

### What does ED syndromic surveillance tell us about acute alcohol harms?

The coded alcohol syndrome represents approximately 0.6% of all ED presentations in NSW. It is known to underestimate the burden of alcohol on ED presentations 4, 6, 18. However, this does not exclude syndromic surveillance as a useful source of data for timely monitoring of acute alcohol harm trends at the state‐wide or local level. This indicator has been used as an outcome measure in relation to legislative and policy changes aimed at reducing harms related to binge drinking, such as reporting on trends in alcohol harm ED presentations associated with the ready‐to‐drink ‘alcopop’ tax and within the Sydney CBD ‘Entertainment Precinct’ 34, 35. It has also been used for reporting purposes, monitoring social drinking trends and related harms and providing situational awareness during mass gatherings, such as Mardi Gras and New Year's Eve celebrations 16, 19, 20, 21. The findings from the current study improve the policy‐relevance of this indicator by informing its appropriate use and interpretation.

Younger age was independently associated with acute harm presentations when compared with chronic harm presentations. This supports the plethora of literature that documents the association between younger age and harms associated with heavy episodic drinking 2, 8, 12, 36. The ED provides an opportunistic setting for ‘teachable moments’ for young people who may not typically seek help. Brief alcohol interventions have been shown to significantly reduce alcohol consumption and alcohol‐related problems among adolescents and young adults and are recommended in the National Alcohol Treatment Guidelines 29, 37.

Emergency departments provide an opportunistic scenario for capturing alcohol‐related injuries. However, systematically identifying alcohol as a contributing factor to injury‐related ED presentations in NSW is not possible 4. As such, ‘late‐night’ arrival to the ED is commonly used as a surrogate measure of alcohol‐related injuries 21, 35, 38. This method assumes that injuries occurring late at night or early in the morning are related to excessive alcohol consumption. Applying these ‘late‐night’ hours to the alcohol syndrome improved the precision of the syndrome in identifying acute harm presentations, supporting the underlying assumption of the method.

### Improving the approach to monitoring acute alcohol harms

Our findings justify consideration of the development of an improved method to accurately identify acute alcohol intoxication harms presenting to the ED using the routine surveillance system. Two potential methods to achieve this goal are described in the succeeding texts.

The first method involves the use of a surrogate measure of acute harms by applying independent predictors of acute harm presentations to the broad alcohol syndrome. This method is based on the already established practice used to estimate alcohol‐related injuries, as discussed in the preceding texts 35, 38, 39. For example, precision to identify acute harm presentations improves to 82% when only selecting alcohol‐coded ED presentations by people aged 12–24 years (Table 1). However, this results in a reduction of the proportion of all acute alcohol harm presentations identified to 42%. Despite this, the improvement in precision enables more accurate monitoring of policy‐relevant trends in acute alcohol harms in young people presenting to the ED.

An alternative approach is to create an acute alcohol sub‐syndrome of the current broad alcohol syndrome. This would entail grouping all acute alcohol intoxication classification codes assigned by the treating physician in the back‐end of the surveillance system and excluding codes related to chronic alcohol problems. Creating a more specific sub‐syndrome risks reducing the accuracy of the grouping due to known variability in coding practices within EDs 32. Post‐hoc analysis showed promising results, with general agreement between the classification based on manual review of the triage notes and the ED diagnosis code assigned for each presentation (Table S3). However, further refining and testing of each method is required before determining a preferred approach.

## Conclusion

There is increasing interest in using administrative datasets for public health research and policy evaluation, given that they can be timely, readily available and relatively inexpensive. The focus of recent policy and legislative changes has been to reduce binge drinking and related harms 34, 40. While the alcohol syndrome in the NSW ED surveillance system has historically been used to indicate the level of acute alcohol harms presenting to the ED, as it currently stands, it includes a substantial component of background noise. The use of additional variables available in the database or the refinement of the underlying codes that are grouped to create the syndrome offer a means of improving the precision of the indicator to more accurately identify acute alcohol harms at the population level and contribute to the ongoing monitoring of trends of acute alcohol harms for policy and program evaluation purposes.

## Supporting information


**Table S1.** Classification codes included in the alcohol syndrome.
**Table S2.** Alcohol harm‐type definitions.
**Table S3.** ED diagnosis, by the classification from the manual review of the triage notes.

Supporting info item
