# Supplementary material for: Diagnosis‐based emergency department alcohol harm surveillance: What can it tell us about acute alcohol harms at the population level?
Source: Drug Alcohol Rev. 2016 Oct 27;35(6):693–701. doi: 10.1111/dar.12458 (PMC5132005; doi:10.1111/dar.12458)
Supplement: Supplementary file 1 — Supporting info item [file DAR-35-693-s001.docx]

**Supplementary Table 1: Classification codes included in the alcohol syndrome**

| **SNOMED-CT** | **ICD-9-CM** | **ICD-10-AM** |
| --- | --- | --- |
| 102612005, 102897001, 135827004, 15167005, 160573003, 160580001, 160581002, 160592001, 160593006, 160599005, 161466001, 163184002, 171208001, 183098002, 183486001, 18653004, 191471000, 191472007, 191473002, 191476005, 191477001, 191478006, 191479003, 191480000, 91481001,191482008, 191802004, 191803009, 191804003, 191805002, 191806001, 191807005, 191809008, 191811004, 191812006, 191813001, 191814007, 191815008, 191882002, 191883007, 191885000, 192206005, 192207001, 192208006, 192209003, 192210008, 192211007, 192212000, 192213005, 192214004, 192215003, 192216002, 198431000000105, 20093000, 2043009, 205351000000101, 207273009, 21000000, 212806006, 212807002, 212808007, 212809004, 212810009, 212811008, 212812001, 212813006, 212814000, 212815004, 212816003, 212817007, 212818002, 212819005, 212820004, 213687005, 216632000, 216633005, 216634004, 216635003, 216636002, 216637006, 216638001, 216639009, 216640006, 216643008, 216644002, 216645001, 216646000, 216648004, 216649007, 216650007, 216652004, 216653009, 219006, 221842002, 221843007, 221844001, 221845000, 221846004, 221847008, 221848003, 221849006, 221850006, 221851005, 221852003, 222103001, 222104007, 222105008, 222106009, 222107000, 222108005, 222110007, 222111006, 222112004, 222113009, 222114003, 222702003, 222703008, 222704002, 222705001, 222706000, 222707009, 222708004, 222709007, 222710002, 222711003, 222713000, 223333005, 223334004, 223335003, 223336002, 223337006, 223338001, 223339009, 223340006, 223341005, 223342003, 223343008, 223344002, 223345001, 223346000, 223347009, 223348004, 223349007, 226136002, 228273003, 228281002, 228312003, 228313008, 228315001, 228316000, 228317009, 228326007, 228330005, 228350009, 228351008, 228353006, 228357007, 228358002, 228364009, 231463001, 231464007, 231465008, 231466009, 231467000, 24165007, 242263000, 242265007, 25702006, 268639004, 268645007, 268683008, 268684002, 268685001, 269765000, 273265007, 273802002, 274257003, 274776000, 28045007, 281004, 281078001, 284591009, 287166006, 29212009, 292880007, 294420000, 300939009, 302237007, 304605000, 304606004, 307730003, 308742005, 311492009, 316322002, 316494009, 32553006, 34938008, 35637008, 361267005, 365967005, 365973006, 371434005, 386449006, 38670004, 408945004, 408946003, 408947007, 408948002, 412198003, 413473000, 415685003, 417096006, 417633001, 419442005, 419572002, 420140004, 42344001, 427013000, 429501006, 429775004, 431260004, 440652002, 441685000, 442766007, 444810000, 445628007, 4953006, 53041004, 53527002, 53936005, 57346004, 61144001, 62213004, 63267000, 64297001, 66590003, 67426006, 6749002, 69482004, 7052005, 7200002, 73097000, 77475008, 78524005, 79578000, 82782008, 85561006, 8635005, 86933000, 87106005, 87460008, 89507002, 95906008 | 291, 291.0–291.5, 291.8–291.9, 303, 303.0, 303.9, 305, 305.0, 535.3, 790.3, 980, E860 | F10, F10.0–F10.9, K29.2, R78.0, T51, T51.0–T51.3, T51.8–T51.9, X45, X65, Y15, Y90, Y90.0–Y90.9, Y91, Y91.0–Y91.3, Y91.9, Z50.2, Z71.4, Z72.1, Z86.41 |

**Note:** If less than five digits are shown in the ICD10 codes, the surveillance system automatically includes any more specific codes in the ICD-10 hierarchy.

ICD-9-CM, International Classification of Diseases 9th revision; ICD-10-AM, International Classification of Diseases 9th revision; SNOMED CT, Systematized Nomenclature of Medicine - Clinical Terminology.

**Supplementary Table 2: Alcohol harm type definitions**

| **Alcohol harm type** | **Definition** |
| --- | --- |
| 1. Acute alcohol intoxication | Includes presentations solely due to the adverse effects of excessive alcohol consumption, defined as presentations that specifically refer to alcohol use or known abbreviations (e.g. ETOH) or detail about type (e.g. wine), amount (e.g. pint) or behaviour (e.g. drunk, drinking), and:   - blood alcohol level greater than 0.05; or - intoxication; - symptoms including strong smell of alcohol on breath, facial flushing, slurred speech, unsteady gait, euphoria, increased activity, volubility, disorderly conduct, slowed reactions, nausea and vomiting, double vision, memory loss, decreased level of consciousness or unconscious, sleepiness, impaired judgement and motor incoordination, insensibility, respiratory failure or coma; - brought in by police for blood and urine test.   Excludes presentations that:   - deny recent consumption of alcohol; - refer to chronic alcohol problems only (as defined below). |
| 1. Chronic alcohol problem | Includes presentations that refer to chronic alcohol misuse as the key presenting problem for the presentation. Chronic alcohol misuse is defined as symptoms of chronic alcohol dependence including:   - Tolerance; - perceived ‘loss of control’ over one’s drinking behaviour; - physical or psychological withdrawal symptoms on cessation of use ; - relief or avoidance of withdrawal symptoms by further drinking; - rapid recommencement of pre-established (high-risk) drinking patterns after a period of abstinence; - indications that the patient is requesting detoxification or rehabilitation for alcohol problems (including ‘detox’ and known detoxification services); - specific mention of a history of alcohol abuse.   Excludes presentations that fall within the ‘acute alcohol intoxication’ category. |
| 1. Acute alcohol intoxication and chronic alcohol problem | Includes presentations that describe both the acute alcohol intoxication category and the chronic alcohol problem category (as defined above). |
| 1. Undetermined | The triage notes do not contain adequate detail to classify the record into one of the categories above. Also includes records that deny recent consumption of alcohol. |

**Supplementary Table 3: ED diagnosis, by the classification from the manual review of the triage notes**

| **ED diagnosis code description** | | **Classification from manual review of the triage text** | | |
| --- | --- | --- | --- | --- |
|  |  | **Acute alcohol harms** | **Chronic alcohol harms** | **Total** |
| Feeling intoxicated | Number | 0 | 1 | 1 |
|  | Percent | 0.0 | 100.0 | 100.0 |
| Alcohol abuse | Number | 29 | 69 | 98 |
|  | Percent | 29.6 | 70.4 | 100.0 |
| Alcohol withdrawal syndrome | Number | 1 | 49 | 50 |
|  | Percent | 2.0 | 98.0 | 100.0 |
| Acute alcoholic intoxication in alcoholism | Number | 10 | 13 | 23 |
|  | Percent | 43.5 | 56.5 | 100.0 |
| Alcoholic gastritis | Number | 13 | 29 | 42 |
|  | Percent | 31.0 | 69.0 | 100.0 |
| Alcohol user | Number | 4 | 14 | 18 |
|  | Percent | 22.2 | 77.8 | 100.0 |
| Binge drinking | Number | 9 | 9 | 18 |
|  | Percent | 50.0 | 50.0 | 100.0 |
| Acute drug intoxication | Number | 18 | 30 | 48 |
|  | Percent | 37.5 | 62.5 | 100.0 |
| Alcohol intoxication | Number | 277 | 101 | 378 |
|  | Percent | 73.3 | 26.7 | 100.0 |
| Accidental poisoning by alcohol | Number | 1 | 0 | 1 |
|  | Percent | 100.0 | 0.0 | 100.0 |
| Finding of alcohol in blood | Number | 2 | 14 | 16 |
|  | Percent | 12.5 | 87.5 | 100.0 |
| Alcohol-induced organic mental disorder | Number | 5 | 3 | 8 |
|  | Percent | 62.5 | 37.5 | 100.0 |
| Acute alcoholic intoxication in alcoholism unspecified drinking behaviour | Number | 6 | 3 | 9 |
|  | Percent | 66.7 | 33.3 | 100.0 |
| Other and unspecified alcohol dependence unspecified drinking behaviour | Number | 0 | 1 | 1 |
|  | Percent | 0.0 | 100.0 | 100.0 |
| Nondependent alcohol abuse unspecified drinking behaviour | Number | 6 | 1 | 7 |
|  | Percent | 85.7 | 14.3 | 100.0 |
| Alcohol withdrawal-induced convulsion | Number | 0 | 1 | 1 |
|  | Percent | 0.0 | 100.0 | 100.0 |
| Alcohol-induced psychosis | Number | 0 | 1 | 1 |
|  | Percent | 0.0 | 100.0 | 100.0 |
| Alcohol dependence | Number | 7 | 35 | 42 |
|  | Percent | 16.7 | 83.3 | 100.0 |
|  |  |  |  |  |
| Toxic effect of alcohol | Number | 2 | 0 | 2 |
|  | Percent | 100.0 | 0.0 | 100.0 |
| Alcohol hallucinosis | Number | 0 | 3 | 3 |
|  | Percent | 0.0 | 100.0 | 100.0 |
| Alcohol poisoning | Number | 4 | 2 | 6 |
|  | Percent | 66.7 | 33.3 | 100.0 |
| Alcohol withdrawal delirium | Number | 0 | 2 | 2 |
|  | Percent | 0.0 | 100.0 | 100.0 |
| Toxic effect of other specified alcohols | Number | 1 | 0 | 1 |
|  | Percent | 100.0 | 0.0 | 100.0 |
| Mental and behavioural disorders due to use of alcohol: Acute intoxication | Number | 119 | 31 | 150 |
|  | Percent | 79.3 | 20.7 | 100.0 |
| Mental and behavioural disorders due to use of alcohol: Dependence syndrome | Number | 12 | 25 | 37 |
|  | Percent | 32.4 | 67.6 | 100.0 |
| Mental and behavioural disorders due to use of alcohol: Psychotic disorder | Number | 0 | 12 | 12 |
|  | Percent | 0.0 | 100.0 | 100.0 |
| Mental and behavioural disorders due to use of alcohol: Unspecified mental and behavioural disorder | Number | 6 | 14 | 20 |
|  | Percent | 30.0 | 70.0 | 100.0 |
| Toxic effect: other alcohols | Number | 1 | 0 | 1 |
|  | Percent | 100.0 | 0.00 | 100.0 |
| Toxic effect: alcohol, unspecified | Number | 0 | 2 | 2 |
|  | Percent | 0.00 | 100.0 | 100.0 |
| Problems related to lifestyle: Alcohol use | Number | 2 | 0 | 2 |
|  | Percent | 100.0 | 0.0 | 100.0 |
| **Total** | **Number** | 535 | 465 | 1000 |

ED, emergency department.
